# Essential leadership skills for health sciences information professionals

**DOI:** 10.29173/jchla29784

**Published:** 2024-12-01

**Authors:** Ashley Jane Leonard

**Affiliations:** Alberta Health Services Calgary, AB, Canada

Crum J, Nunez AV, editors. **Essential leadership skills for health sciences information professionals**. Lanham, MD: Rowman & Littlefield; 2023. Softcover: 184 p. ISBN: 978-1-5381-6823-3. Price: USD$79.00. Available from: https://rowman.com/ISBN/9781538168233/Essential-Leadership-Skills-for-Health-Sciences-Information-Professionals

Strong leadership skills are crucial to the management of a successful, well run library. While it is not uncommon for librarians to have taken classes or modules on leadership as part of their schooling, the breadth and depth (and perhaps currency) of that information is likely not comprehensive enough for aspiring library managers to confidently step into a leadership role. *Essential leadership skills for health sciences information professionals* aims to fill this gap by providing readers with an accessible and quick-to-read guide on the topic of all things library leadership. As the title suggests, the book is specifically geared towards those working within a health sciences library context. Over the course of 11 chapters, Crum and Nunez share their knowledge on various topics relating to leadership; this is done in conjunction with 12 other library leaders, namely those in the roles of library deans, directors, and vice presidents.

The book is divided into three sections. Section one, which comprises of two chapters, is concerned with topics relating to leadership that have an external library focus. Chapter one highlights the importance of being aware of the context in which the reader's library exists, while understanding that this may look different depending on the kind of health sciences library the reader works in. Closely linked, chapter two focuses on the identification of external stakeholders and building relationships with them.

Section two then turns its attention inward and looks at the process of leading library teams. Four chapters are dedicated to this section. Chapter three focuses on setting direction and provides information regarding the all important task of creating a strategic plan for the reader's library. Following on from that, chapter four looks at the process of building a successful team. The chapter explores the hiring process, employee retention, and workplace morale among other topics. The topic of staff engagement is covered in chapter five; topics such as engagement throughout the employee lifecycle including recruiting, onboarding, and how to manage for employee engagement are covered. The last chapter in this section, chapter six, looks at the very timely topic of diversity, equity, and inclusion through an anti racist lens. The Joint ALA/ARL Building Cultural Proficiencies for Racial Equity Framework Task Force publication, “Cultural proficiencies for racial equity: a framework” [1], informs the information included in this chapter.

Lastly, section three looks at the specific tools, techniques, and growth opportunities that library leaders may find useful to consider. Chapter seven looks at the topic of project management, including several tools that are available to help structure and guide this work. Chapter eight focuses on change management; various change management models are discussed, as are other important elements relating to this topic including feedback and transparency. Decision making and problem solving make up the focus for chapter nine, including a look at how to improve decision making from an organizational perspective. Chapter 10 examines crises management and provides topics that a leader may want to consider before a crisis arises, as well as during one. The last chapter, chapter 11, is focused on self care from a management perspective, an oft overlooked aspect of leadership. The chapters are fairly short in length and within each of these chapters, content is further divided into bolded subtopics which makes for quick reading. The strength of this book lies in the reference lists that are included at the end of every chapter. Readers are provided with a wealth of additional resources to consult as the need arises; both online and in print resources are provided, with website URLs included where appropriate.

New or early career library leaders will most likely benefit from the information contained in this book, although seasoned leaders may also find it a nice refresh or a concise way to learn about current library topics such as diversity, equity, and inclusion. The book is written in a very accessible way and jargon is limited. Given the broad span of topics included, some readers may find that the material that is covered is not as comprehensive as they might like – but in my opinion, this book serves more as a ‘leadership primer’, rather than a deep dive on the topic. It succinctly provides leaders with information on topics to be aware of, and importantly, a place to start exploring those topics. If readers are wanting more information, the reference lists provide a good next set of resources to consult. I would recommend this book to anyone hoping to get an overview of some of the most important aspects of library leadership, regardless of where they find themselves in their career journey.
